# Risk of vascular diseases in patients with dermatitis herpetiformis and coeliac disease: a long-term cohort study

**DOI:** 10.1080/07853890.2023.2227423

**Published:** 2023-06-28

**Authors:** Noora Nilsson, Joonas Leivo, Pekka Collin, Inka Koskinen, Katri Kaukinen, Heini Huhtala, Johanna Palmio, Timo Reunala, Kaisa Hervonen, Teea Salmi, Camilla Pasternack

**Affiliations:** a Celiac Disease Research Center, Faculty of Medicine and Health Technology, Tampere University, Tampere, Finland; b Department of Dermatology, Tampere University Hospital, Tampere, Finland; c Heart Hospital, Tampere University Hospital, Tampere, Finland; d Department of Gastroenterology and Alimentary Tract Surgery, Tampere University Hospital, Tampere, Finland; e Department of Gastroenterology, Hospital Nova of Central Finland, Jyväskylä, Finland; f Department of Internal Medicine, Tampere University Hospital, Tampere, Finland; g Faculty of Health Sciences, Tampere University, Tampere, Finland; h Department of Neurology, Tampere University and Tampere University Hospital, Tampere, Finland

**Keywords:** Dermatitis herpetiformis, coeliac disease, gluten-free diet, vascular diseases, cardiovascular disease, ischaemic heart disease, cerebrovascular disease, thromboembolic disease

## Abstract

**Introduction:**

Dermatitis herpetiformis (DH) is a cutaneous manifestation of coeliac disease. Increased cardiovascular morbidity has been reported in coeliac disease, but in DH only little is known about this. In this cohort study with a long-term follow-up, the risk for vascular diseases in patients with dermatitis herpetiformis (DH) and coeliac disease was assessed.

**Methods:**

The study consisted of 368 DH and 1072 coeliac disease patients with biopsy-proven diagnosis performed between 1966 and 2000. For each DH and coeliac disease patient three matched reference individuals were obtained from the population register. Data regarding all outpatient and inpatient treatment periods between 1970 and 2015 were reviewed for diagnostic codes of vascular diseases from the Care Register for Health Care. Cox proportional hazard model was used to assess the risks for the diseases studied and the HRs were adjusted for diabetes mellitus (aHR).

**Results:**

The median follow-up time of DH and coeliac disease patients was 46 years. The risk for cardiovascular diseases did not differ between DH patients and their references (aHR 1.16, 95% CI 0.91–1.47), but among coeliac disease patients, the risk was increased (aHR 1.36, 95% CI 1.16–1.59). The risk for cerebrovascular diseases was found to be decreased in DH patients when compared with references (aHR 0.68, 95% CI 0.47–0.99) and increased in coeliac disease patients (aHR 1.33, 95% CI 1.07–1.66). The risk for venous thrombosis was increased in coeliac disease patients (aHR 1.62, 95% CI 1.22–2.16) but not in DH.

**Conclusions:**

The risk for vascular complications appears to differ between DH and coeliac disease. In DH the risk for cerebrovascular diseases seems to be decreased, while in coeliac disease an elevated risk for cerebrovascular and cardiovascular diseases was observed. These differing vascular risk profiles between the two manifestations of the same disease merit further investigation.

## KEY MESSAGE

- An increased risk for cardiovascular diseases was observed among patients with coeliac disease, but not among patients with dermatitis herpetiformis, a cutaneous manifestation of coeliac disease.
- The risk for cerebrovascular diseases was shown to be decreased in dermatitis herpetiformis patients, but conversely, an increased risk for cerebrovascular diseases was identified in coeliac disease patients.
- Coeliac disease, but not dermatitis herpetiformis, was shown to be associated with increased risk for venous thrombosis.

## Introduction

Coeliac disease is a common immune-mediated intestinal disorder driven by dietary gluten in genetically predisposed individuals [1]. Gastrointestinal symptoms, such as diarrhoea and abdominal pain, are considered to be the classic symptoms of coeliac disease, but the disease may also present with a variety of extraintestinal symptoms [1]. Dermatitis herpetiformis (DH), a cutaneous manifestation of coeliac disease, typically presents as a pruritic, blistering, and papular rash [2]. In DH patients, granular immunoglobulin A (IgA) deposits are seen in the papillary dermis and epidermal transglutaminase (TG3) acts as the target autoantigen [3]. In addition, patients with DH also have a coeliac-type systemic disease with circulating antibodies against tissue transglutaminase (TG2) and a varying degree of enteropathy, ranging from minor inflammatory changes to severe villous atrophy in the small bowel mucosa [2]. Regardless of the intestinal findings, DH patients rarely have severe gastrointestinal symptoms [2].

The treatment for all phenotypes of coeliac disease, including DH, is a lifelong gluten-free diet (GFD). Strict adherence to the diet eventually normalizes the serology, heals the mucosal damage, and leads to resolution of the symptoms deriving from both the skin and the intestine [1,2]. Also, in those with severe DH rash, additional treatment with dapsone medication is needed when starting the GFD [2]. Adherence to GFD has additionally important prognostic significance as it has been shown to protect against lymphoma and bone fractures, possible complications of both coeliac disease and DH [4,5]. However, there is evidence suggesting that the long-term prognosis of patients with DH and coeliac disease differs in some respects, since patients with DH have shown to be at lower risk for bone complications and renal comorbidities than patients with other phenotypes of coeliac disease [6,7]. Mortality among coeliac disease patients has moreover been shown in several studies to be increased [8], while in DH there are few studies indicating lower mortality rates than in general population [9–11]. Coeliac disease has been associated with certain vascular diseases, such as ischaemic heart disease, stroke, and thromboembolic complications [12,13], which may partly account for the increased mortality seen among patients with coeliac disease [9,14]. However, the data on vascular diseases associated with DH are very limited [13], but, intriguingly, the decreased mortality rates seen in DH patients have been associated especially with lowered cardiovascular and cerebrovascular mortality [9–11].

This study aimed to assess for the first time the risk of vascular diseases in a biopsy-proven cohort of patients with DH with long-term follow-up using matched reference individuals and nationwide hospital discharge register as a data source. In addition, using a large cohort of coeliac disease patients, the risk of vascular diseases was compared between DH and coeliac disease patients.

## Materials and methods

### Patients and reference individuals

All patients diagnosed with DH in the Tampere University Hospital catchment area between the years 1969 and 2000 (*n* = 394) were initially included in the study. In each patient, the diagnosis had been based on granular IgA deposits seen in the papillary dermis in direct immunofluorescence together with a typical clinical picture. However, DH patients diagnosed with coeliac disease more than one year prior to DH diagnosis were excluded from the study (*n* = 26) and thus the final DH study group consisted of 368 patients.

According to the national guidelines of the time, all the DH patients in the study had been recommended to undergo gastroscopy with small bowel biopsy obtainment at the time of DH diagnosis and morphology was graded by an experienced pathologist as subtotal or partial villous atrophy or normal mucosa. After diagnosis, all patients had been advised to adhere to a strict GFD and a visit to a dietitian was recommended. Dapsone medication had been started according to routine policy for those with severe rash, approximately on 70% of the patients [11]. After being diagnosed, patients were followed up at the special outpatient clinic until rash had resolved and dapsone could be discontinued.

Coeliac disease patients diagnosed in the Tampere University Hospital catchment area between 1966 and 2000 (*n* = 1076) were also enrolled in the study. In all patients, coeliac disease had been diagnosed based on typical histologic findings, i.e. duodenal villous atrophy and crypt hyperplasia. The coeliac disease patients’ discharge register data (see below) were reviewed regarding the presence of International Classification of Diseases (ICD)-8–10 codes corresponding to DH (693.99, 694.0 A, L13.0), and the medical records of those patients with corresponding codes were reviewed in case of false diagnoses. Four coeliac disease patients were excluded from the study because DH diagnosis could not be ruled out. The final number of coeliac disease patients included in the study was thus 1072. After diagnosis, all coeliac disease patients had been instructed to begin a strict GFD, and dietary guidance by a dietitian was recommended. Depending on the severity of the disease and the policies of the time, follow-up was continued in primary or tertiary health care.

As references, three reference individuals matched for age, sex, calendar year, and place of residence were chosen from the population register maintained by the Digital and Population Data Services Agency in Finland for each DH and coeliac disease patient. The date of the index patient’s DH or coeliac disease diagnosis was used as the index date for matching. From the reference group, the individuals with ICD-8–10 codes corresponding to coeliac disease (269, 579.0 A, K90.0) or DH (693.99, 694.0 A, L13.0) in the discharge register were excluded from the study. Five reference individuals from the DH reference group and 19 references from the coeliac disease reference group were excluded because of DH or coeliac disease diagnoses. After exclusion, the eventual number of the DH reference group was 1099 and the number of the coeliac disease reference group was 3197.

### Study protocol

Data regarding the morbidity of DH and coeliac disease patients and references were recorded in the Care Register for Health Care. This is a mandatory national healthcare register maintained by the Finnish Institute for Health and Welfare, which contains data on hospital admissions and discharge days and causes of hospitalization recorded with ICD codes since 1969. Data on specialized outpatient care have been recorded in the Care Register since 1994. All the outpatient and inpatient treatment periods between 1 January 1970, and 31 December 2015, were noted. The primary outcomes of the study were the specific diagnoses of vascular diseases and ICD codes matching these diseases recorded in the Care Register for Health Care were used to collect the data (See Supplementary Table 1). The diseases studied were categorized into four groups: cardiovascular diseases, cerebrovascular diseases, vascular diseases of the aorta and peripheral arteries, and vein thromboses. In the study the group of cardiovascular diseases was defined to include ICD codes matching coronary artery disease, atherosclerotic valve disease, and heart failure, while cerebrovascular diseases included transient ischaemic attacks (TIA) and strokes of ischaemic or thromboembolic origin. As possible confounding covariates, the ICD codes matching hypertension, atrial fibrillation, dia­betes mellitus (types 1 and 2), hypercholesterolaemia, chronic obstructive pulmonary disease, and sleep apnoea were identified.

The dates of death and emigration of the DH and coeliac disease study patients and references were obtained from the Population Register of Finland. In addition, data on the findings of small bowel histopathology of coeliac disease and DH patients at diagnosis were gathered from the medical records.

The study protocol was approved by the Regional Ethics Committee of Tampere University Hospital (R16090) and followed the ethical principles of the Helsinki Declaration. As the study was register based, no consent from patients was required.

### Statistical analysis

Incidence rates for the first diagnoses of specific vascular diseases were calculated, and using Cox proportional hazard models, hazard ratios (HR), and 95% confidence intervals (CI) for the different vascular diseases were estimated. Kaplan-Meier was used for the failure curves. Follow-up started on 1 January 1970, or if the patient or reference was born after that, from the date of birth. Follow-up ended at the first occurrence of the outcome measure analyzed, emigration, death, or December 2015, whichever came first. Concerning possible confounding comorbidities, the differences between patients and matched reference individuals were evaluated using conditional logistic regression, and the main results were consequently further adjusted for diabetes mellitus (adjusted hazard ratio, aHR), and also for atrial fibrillation in coeliac disease patients.

In DH patients, the association between the cerebrovascular and cardiovascular diseases studied and small bowel histology at the time of diagnosis were analyzed. When performing the analyses, the mucosal histopathology was classified as normal mucosa or villous atrophy, and the variables were adjusted for sex and age at the end of follow-up.

All the statistical analyses were performed in cooperation with a statistician, using SPSS version 26 (IBM SPSS Statistics for Windows, Version 26.0. Armonk, NY: IBM Corp. USA).

## Results

The median follow-up time in both study groups, DH and coeliac disease, was 46 years (Table 1). DH patients were more often male (51%) and slightly older at the end of follow-up when compared with coeliac disease patients, but median age at DH or coeliac disease diagnosis did not differ. Among DH patients, 71% had small bowel villous atrophy and 29% normal villous architecture at diagnosis, while all coeliac disease patients had villous atrophy by definition. Diabetes mellitus and atrial fibrillation were found to be significantly more common among coeliac disease patients than among their references, while in DH patients, diabetes mellitus was seen less frequently than in the reference population and there was no significant difference regarding the incidence of atrial fibrillation. The incidences of other comorbidities studied did not significantly differ between the study groups and references (Table 1).

**Table 1.** Demographic data and studied comorbidities of dermatitis herpetiformis (DH) and coeliac disease patients and their matched references.

	DH		Coeliac disease	
	Patients	References	Patients	References
Total, *n*	368	1099	1072	3197
Females, *n* (%)	180 (49)	539 (49)	729 (68)	2174 (68)
Age at time of DH or coeliac disease diagnosis, median (IQR), years	39 (5–84)	39 (5–84)	39 (0–85)	39 (0–85)
Age at time of DH or coeliac disease diagnosis, *n* (%)				
Under 18 years	18 (5)	53 (5)	232 (22)	691 (22)
18–40 years	173 (47)	517 (47)	338 (32)	1005 (32)
Over 40 years	177 (48)	529 (48)	502 (47)	1501 (47)
Year of DH or coeliac disease diagnosis, *n* (%)				
Before 1980	87 (24)	259 (24)	110 (10)	329 (10)
Between 1981 and 1990	187 (51)	559 (51)	441 (41)	1314 (41)
Between 1991 and 2001	94 (26)	281 (26)	521 (49)	1554 (49)
Person-years of follow-up	14 883	43 197	42 289	129 013
Follow-up time, median (IQR), years	46 (43–46)	46 (39–46)	46 (39–46)	46 (39–46)
Age at end of follow-up, median (IQR), years	68 (23–96)	67 (17–101)	62 (9–97)	62 (6–100)
Comorbidities, *n* (%)				
Diabetes mellitus	29 (8)^a^	132 (12)	115 (11)^a^	248 (8)
Hypertension	69 (19)	239 (22)	186 (17)	508 (16)
Atrial fibrillation	47 (13)	147 (13)	120 (11)^a^	285 (9)
Hypercholesterolemia	12 (3)	53 (5)	38 (4)	88 (3)
Sleep apnoea	16 (4)	60 (6)	32 (3)	108 (3)
COPD	3 (1)	8 (1)	7 (1)	16 (1)

IQR: interquartile range; COPD: chronic obstructive pulmonary disease.

^a^Statistically significant difference when compared to references.

**Figure 1.**
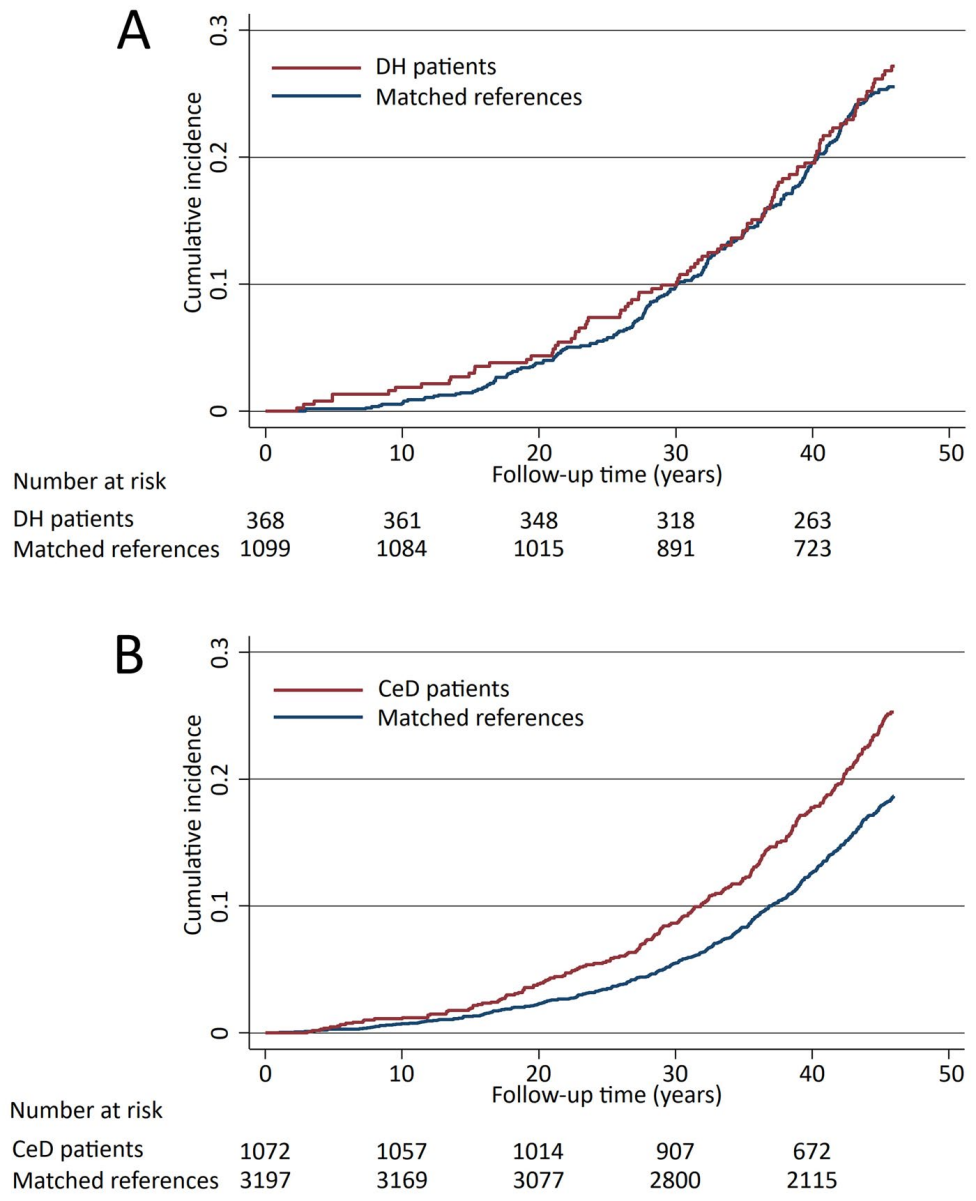
Kaplan-Meier failure curves estimating the cumulative incidence of the cardiovascular diseases studied in dermatitis herpetiformis (DH) patients (A, diabetes mellitus adjusted hazard ratio 1.16; 95% CI 0.91–1.47) and coeliac disease (CeD) patients (B, diabetes mellitus adjusted hazard ratio 1.36; 95% CI 1.17–1.59).

The risk of all cardiovascular diseases studied for the whole follow-up time did not differ between the DH patients and their references (aHR 1.16; 95% CI 0.91–1.47) (Figure 1 and Table 2). Nor were significant difference detected when specific cardiovascular diseases were analyzed separately, except for atherosclerotic valve disease being more common in the DH patient group (Table 2). Also, the risk for acute ischaemic events was found not to differ significantly among DH patients when compared with their references (aHR 1.26; 95% CI 0.87–1.81). Among coeliac disease patients, the risk of all cardiovascular diseases studied was significantly higher than among their references (aHR, 1.36; 95% CI 1.17–1.59) (Figure 1 and Table 2). More specifically, coeliac disease patients were shown to have increased risks for coronary artery disease and atherosclerotic valve disease (Table 2). A slightly increased risk for acute ischaemic events was also seen in the coeliac disease patient group (aHR 1.27; 95% CI 0.99–1.63).

**Table 2.** Incidence rates and unadjusted and adjusted hazard ratios (HR) with 95% confidence intervals (CI) for different vascular diseases in dermatitis herpetiformis (DH) and coeliac disease patients and their matched references during the total follow-up time.

	Patients		References					
	Events	Incidence/10,000 PY	Events	Incidence/10,000 PY		HR	95% CI	Adjusted HR^a^	95% CI
All cardiovascular diseases studied								
DH	93	62.49	246	56.95	1.07	0.84–1.36	1.16	0.91–1.47
Coeliac disease	229	54.15	497	38.52	1.43	1.22–1.67	1.36	1.17–1.59
Coronary artery disease								
DH	73	48.79	190	43.62	1.09	0.83–1.43	1.17	0.90–1.54
Coeliac disease	189	44.34	408	31.48	1.43	1.20–1.70	1.36	1.14–1.62
Atherosclerotic valve disease								
DH	10	6.41	12	2.66	2.34	1.01–5.43	2.54	1.10–5.92
Coeliac disease	21	4.73	22	1.65	2.90	1.60–5.28	2.86	1.57–5.19
Heart failure								
DH	41	26.42	119	26.70	0.97	0.68–1.38	1.06	0.74–1.51
Coeliac disease	91	20.70	211	15.95	1.30	1.02–1.68	1.24	0.97–1.58
All cerebrovascular diseases studied								
DH	33	21.39	140	31.63	0.66	0.45–0.96	0.68	0.47–0.99
Coeliac disease	115	26.39	255	19.39	1.38	1.11–1.72	1.33	1.07–1.66
Transient ischemic attack (TIA)								
DH	13	8.36	42	9.36	0.87	0.47–1.62	0.89	0.48–1.66
Coeliac disease	55	12.48	96	7.24	1.74	1.25–2.42	1.71	1.23–2.39
Ischemic or thromboembolic stroke								
DH	25	16.10	113	25.40	0.61	0.40–0.95	0.65	0.42–0.99
Coeliac disease	75	17.06	181	13.68	1.26	0.96–1.65	1.20	0.92–1.57
All vascular diseases of aorta and peripheral arteries studied								
DH	15	9.62	51	11.35	0.83	0.46–1.47	0.88	0.49–1.56
Coeliac disease	43	9.73	11	8.36	1.17	0.82–1.66	1.09	0.77–1.55
Atherosclerosis of aorta and peripheral arteries								
DH	11	7.05	37	8.22	0.84	0.43–1.64	0.90	0.46–1.76
Coeliac disease	35	7.91	79	5.94	1.34	0.90–1.99	1.22	0.82–1.82
Aneurysm/ Dissecation of aorta and peripheral arteries								
DH	3	1.92	13	2.88	0.65	0.19–2.27	0.67	0.19–2.35
Coeliac disease	9	2.02	33	2.48	0.82	0.39–1.71	0.81	0.22–1.23
Occlusion/embolism of aorta and peripheral arteries								
DH	1	0.64	7	1.55	0.40	0.05–3.25	0.43	0.05–3.48
Coeliac disease	9	2.02	20	1.50	1.35	0.62–2.97	1.24	0.56–2.73
All vein thromboses studied								
DH	20	12.86	57	12.76	0.98	0.59–1.64	1.04	0.62–1.73
Coeliac disease	74	16.89	135	10.29	1.68	1.26–2.22	1.62	1.22–2.16
Pulmonary embolism								
DH	4	2.56	20	4.43	0.56	0.19–1.64	0.62	0.21–1.81
Coeliac disease	21	4.73	46	3.61	1.38	0.82–2.30	1.28	0.77–2.15
Deep vein thrombosis								
DH	14	8.99	33	7.36	1.20	0.64–2.24	1.24	0.66–2.33
Coeliac disease	49	11.15	91	6.87	1.64	1.16–2.32	1.61	1.14–2.28
Other vein thrombosis								
DH	3	1.92	11	2.44	0.76	0.21–2.73	0.80	0.22–2.86
Coeliac disease	17	3.83	15	1.12	3.43	1.72–6.88	3.38	1.69–6.76

^a^Adjusted for diabetes mellitus.

PY: Person years.

**Figure 2.**
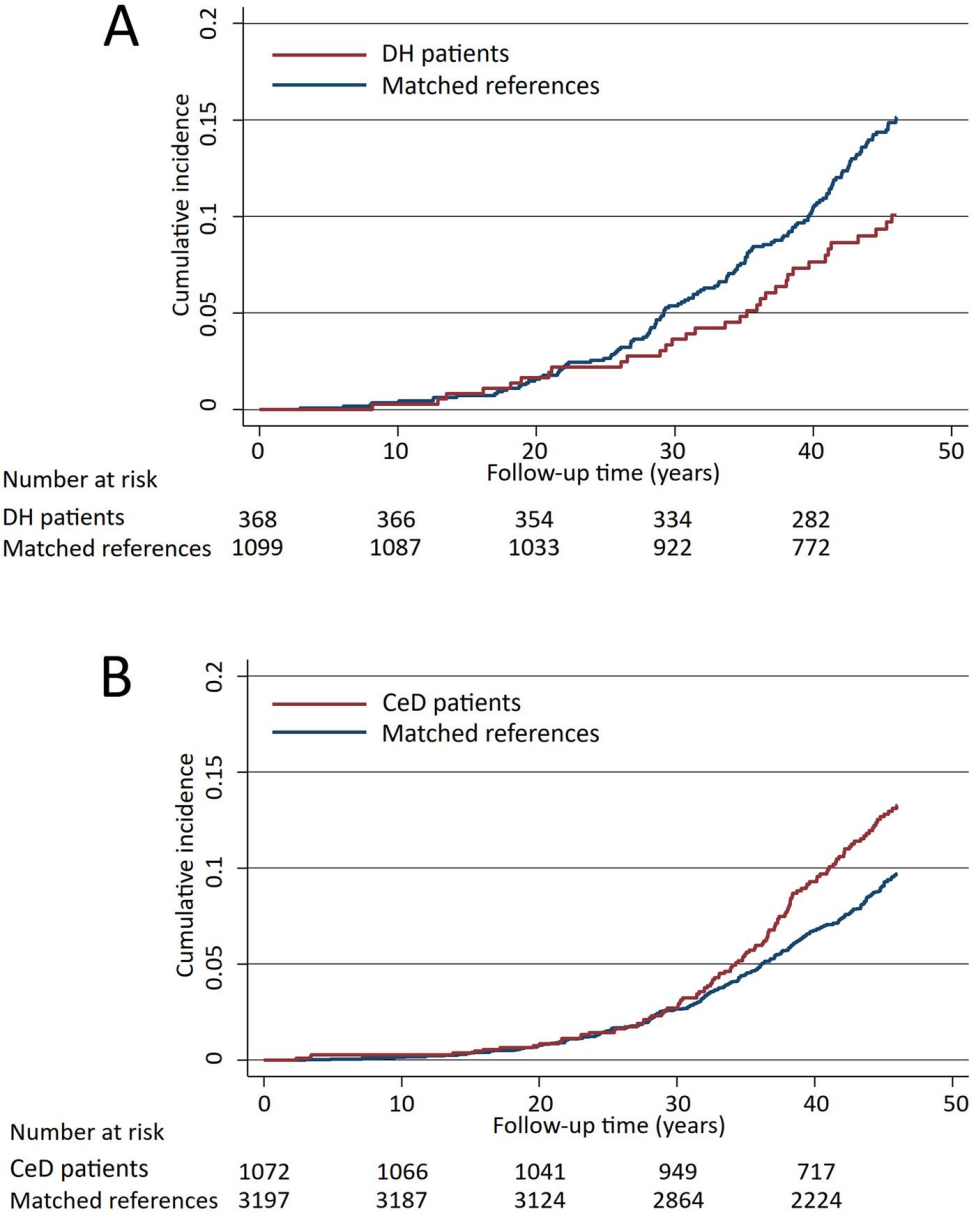
Kaplan-Meier failure curves estimating the cumulative incidence of all cerebrovascular outcomes studied in dermatitis herpetiformis (DH) patients (A, diabetes mellitus adjusted hazard ratio 0.68; 95% CI 0.47–0.99) and coeliac disease (CeD) patients (B, diabetes mellitus adjusted hazard ratio 1.33; 95% CI 1.07–1.66).

Regarding all cerebrovascular diseases studied, the risk was found to be decreased in DH patients compared with references (aHR 0.68; 95% CI 0.47–0.99) (Figure 2 and Table 2), and specifically the risk for stroke was decreased in DH patients (Table 2). In contrast to DH, among coeliac disease patients an increased risk for cerebrovascular diseases in general (aHR 1.33; 95% CI 1.07–1.66) as well as for transient ischaemic attacks (TIA) were seen when compared with references (Figure 1 and Table 2).

No significant difference in the risk for venous thrombosis was seen in DH, but in coeliac disease the risks for venous thrombosis altogether as well as for deep vein thrombosis and other vein thromboses were increased (Table 2). No significant increases in the risks for atherosclerotic diseases of the aorta and peripheral arteries in DH or coeliac disease patients could be demonstrated (Table 2).

In addition to diabetes mellitus, all the above-mentioned analyses regarding the coeliac disease study group were also adjusted for atrial fibrillation, since this was found to be more prevalent in the coeliac disease group than among the references (Table 1). However, risk estimates were not significantly influenced, and were therefore excluded from the results. Furthermore, when analyzing the association of small bowel histology at diagnosis and the vascular morbidities in DH patients, it was found that DH patients having villous atrophy at diagnosis were not at greater risk of cardiovascular or cerebrovascular diseases than were those without villous atrophy at diagnosis after adjusting for sex and age at the end of follow-up (HR 1.32; 95% CI 0.79–2.19 and HR 0.89; 95% CI 0.39–2.01 respectively).

## Discussion

In this study including large and well-defined cohorts of DH and coeliac disease patients, the risk for cerebrovascular diseases was found to be decreased in DH patients, while the risk for other vascular diseases did not differ from that of references. On the contrary, coeliac disease was associated with an increased risk for cardiovascular diseases, cerebrovascular diseases, and vein thrombosis.

The risk for vascular diseases in DH has so far been only rarely studied [13]. The existing evidence is mostly from DH mortality studies, in which lower cardio- and cerebrovascular mortality has been reported [10,11,13]. The results of the present study corroborate those of our earlier DH mortality study with overlapping study cohorts [11], demonstrating significantly reduced cerebrovascular mortality among 476 patients with DH, while mortality due to ischaemic heart disease was similar to that in matched general population. Nevertheless, reduced mortality from ischaemic heart disease has also been reported in a British hospital-based series of 152 DH patients [10].

In coeliac disease, an increased risk for vascular diseases has previously been reported, but the existing evidence is not entirely consistent [13]. Nationwide cohort studies from Sweden have demonstrated increased risks for ischaemic heart disease and stroke in coeliac disease [15–17], while in a British population-based study coeliac disease patients were not found to be at elevated risk for stroke or myocardial infarction [18]. Nonetheless, in a large cohort study [14] and a UK biobank study [19] assessing cause-specific mortality among coeliac disease patients, mortality due to cardiovascular disease was reportedly higher in patients with coeliac disease than in general population. Accordingly, a coeliac disease mortality study from Tampere [9] demonstrated elevated mortality rates in ischaemic heart disease, however, quite surprisingly, decreased cerebrovascular mortality was seen in coeliac disease patients. In addition, there are some more recent coeliac disease mortality studies from Finland, in which the cardiovascular mortality among coeliac disease patients were not increased, however these studies also included DH patients [20,21].

In the present study no increased risk for venous thrombosis was seen in DH, and to the best of our knowledge, no prior studies are available for purposes of comparison. However, an excess risk of thromboembolic complications in coeliac disease was seen in this study, which concurs with some prior evidence [12]. Nationwide register studies from Sweden have demonstrated a positive association between coeliac disease and venous thromboembolism [22] and pulmonary embolisms [23]. However, no such association was discovered in a Danish study [24].

The differences observed in the vascular risk profiles between DH and coeliac disease in this study is rather intriguing, DH being a phenotype of coeliac disease. However, DH patients differ from patients with other phenotypes of coeliac disease, for example, by being more often seronegative and by having less severe intestinal damage [2]. These differences could at least partly explain the disparity detected in vascular morbidities, especially as it has been proposed that the chronic systemic inflammation in coeliac disease predisposes patients to atherosclerosis [25], and decreased levels of nutrients caused by malabsorption have also been linked to elevated cardiovascular risk [26,27]. The present study, however, found no association between the severity of intestinal damage at the time of DH diagnosis with vascular diseases, nor has any such association been detected in coeliac disease [15]. Likewise, in mortality studies conducted on DH and coeliac disease patients, no significant association between the degree of small bowel mucosal damage and mortality has been established either [11,28]. Thus, the main explanation for the differences seen in vascular disease risk profiles between DH and other phenotypes of coeliac disease is likely elsewhere. The positive impact of strict GFD on the vascular function of coeliac disease patients have also been proposed [29], but there is no consistent evidence of disparity in dietary adherence between the different phenotypes [30]. The only distinct difference in the treatment of the two different phenotypes of coeliac disease is dapsone medication, which has anti-inflammatory effects and in most cases is used in DH as an additional treatment. However, dapsone is discontinued on average two years after GFD is started [2], so its significance regarding cardiovascular morbidities is improbable.

In this register study not all known cardiovascular risk factors could be studied, but it was found that the incidences of hypercholesterolaemia, hypertension, sleep apnoea and chronic obstructive pulmonary disease did not significantly differ between DH or coeliac patients when compared with their references. However, according to the questionnaire survey executed as part of the prior DH mortality study with overlapping DH cohort [11], DH patients had less self-reported hypercholesterolaemia and present and past smoking than their matched controls, and while reported use of alcohol did not differ significantly, fewer alcohol related deaths were seen in patients with DH, indicating lesser heavy consumption of alcohol. Nevertheless, somewhat similar findings regarding hypercholesterolaemia [31] and smoking [32] have also been made concerning patients with coeliac disease, suggesting that the reason for the increased risk is elsewhere. Also, as a risk factor for thrombosis, there is some evidence of thromboembolic autoantibodies in coeliac disease [33]. However, in a cohort study from Finland [34] coeliac disease patients with different phenotypes were found to have elevated levels of antiphospholipid antibodies, and the levels did not differ between different phenotypes, thereby indicating that the aetiology of thrombotic events is more complex.

A notable strength in this study was the large cohorts of DH and coeliac disease patients with biopsy-proven diagnoses and a long follow-up time. The study was also conducted in an area with very high GFD adherence rates, with the percentage of DH and coeliac disease patients adhering to strict GFD varying between 72–88% in earlier studies conducted in the area [11,35]. Moreover, comparisons were made with a matched reference population. As a limitation, not all possible confounding factors, such as smoking, socioeconomic status, or body mass index, could be considered. Furthermore, the diagnoses of vascular diseases were gathered from Special Health Care registers, and were not verified by assessing medical records, thus are not fully comprehensive. However, in relation to the data, one can assume that the different study groups were comparable. Also, the main source of register data used in the study, the Finnish Hospital Discharge Register, is considered to be a rather reliable source of data, as the positive predictive values of certain cerebrovascular and cardiovascular disease diagnoses reported in the register have been shown to vary between 85–97% [36].

## Conclusions

In this cohort study with a long follow-up time, the risks for vascular diseases were shown to differ between DH and coeliac disease. In DH reduced risk for cerebrovascular morbidity was detected while in coeliac disease the risk for cardio- and cerebrovascular diseases was increased. The degree of small bowel mucosal damage does not, at least by itself, seem to account for the vascular morbidity risk. Thus, the explanation behind the differences seen in the vascular morbidities between the two phenotypes of coeliac disease is unclear and further research is warranted.

## Supplementary Material

Supplemental Material

Supplemental Material

Supplemental Material

## Data Availability

Due to the nature of this research, participants of this study did not agree to their data being shared publicly, so supporting data is not available.
