# Supplementary material for: Risk of vascular diseases in patients with dermatitis herpetiformis and coeliac disease: a long-term cohort study
Source: Ann Med. 2023 Jun 28;55(1):2227423. doi: 10.1080/07853890.2023.2227423 (PMC10308869; doi:10.1080/07853890.2023.2227423)
Supplement: Supplemental Material [file IANN_A_2227423_SM7173.docx]

**Supplementary Table 1.** International Classification of Diseases (ICD) codes used for outcomes and comorbidities. ICD-8 coding was used in the years 1969–1986, ICD-9 between 1987 and 1995, and ICD-10 from 1996 onwards.

|  |  | | ICD-10 | ICD-9 | ICD-8 |
| --- | --- | --- | --- | --- | --- |
| ATHEROSCLEROTIC HEART DISEASES | | |  |  |  |
|  | Coronary artery disease | | I20-I23, I25 | 410, 411.0, 411.1, 413, 414.0, 412.0 | 410–414 |
|  |  | Acute ischemic events | I20-I23 | 410, 411.0 | 410–411 |
|  | Atherosclerotic valve disease | | I35.0, I35.2 | 424.1C, 424.1B | 424.1 |
|  | Heart failure | | I50 | 414.8, 402 | 427.00, 402 |
| CEREBROVASCULAR DISEASES | | |  |  |  |
|  | Cerebral artery atherosclerosis | | I67.2 | 437.0A | 437 |
|  | Transient ischemic attack (TIA) | | G45 | 453 | 435 |
|  | Ischemic or thromboembolic stroke (including retinal artery occlusion) | | I63, I64, H34 | 433.*A, 434.*A, 436362.3A, 362.3B | 432–434, 436 |
|  | Cerebral artery occlusion without stroke | | I65-I66 | 433.*X, 434.9X, 437.1A | 400.20, 438 |
| DISEASES OF AORTA AND PERIPHERAL ARTERIES | | |  |  |  |
|  | Atherosclerosis | | I70.0, I70.1, I70.2, I70.8, I70.9 | 440.0, 440.1, 440.2, 440.8, 440.9 | 440.0, 440.1, 440.2, 440.3, 440.9, 443.9 |
|  | Aneurysm/dissecation | | I71, I72 | 441, 442 | 441, 442 |
|  | Occlusion/embolism | | I74.0, I74.1 | 444.0, 444.1 | 444.0, 444.1 |
|  |  | | I74.2, I74.3, I74.4, I74.5, I74.8, I74.9 | 444.2A, 444.8A, 444.8X, 444.9X | 444.2, 444.3, 444.4, 444.9 |
| VEIN THROMBOSES | | |  |  |  |
|  | Pulmonary embolism | | I26 | 415 | 450 |
|  | Deep vein thromboses | | I80, I81 | 451.1, 452 | 451.0, 452 |
|  | Other vein thromboses | | I82 | 453, 451.0A, 451.2A | 453 |
| COMORBIDITIES | | |  |  |  |
|  | Hypertension | | I10 | 410 | 410 |
|  | Atrial fibrillation | | I48 | 427.3A | 427.92 |
|  | Hypercholesterolemia | | E78 | 272.0A, 272.0X | 272, 279 |
|  | Diabetes mellitus (DM) | | E10, E11 | 250A, 250B | 250 |
|  | COPD | | J44.9 | 496 | 491.04 |
|  | Sleep apnea | | G47.3 |  |  |
