# Supplementary figures and images for: Risk of vascular diseases in patients with dermatitis herpetiformis and coeliac disease: a long-term cohort study
Source: Ann Med. 2023 Jun 28;55(1):2227423. doi: 10.1080/07853890.2023.2227423 (PMC10308869; doi:10.1080/07853890.2023.2227423)

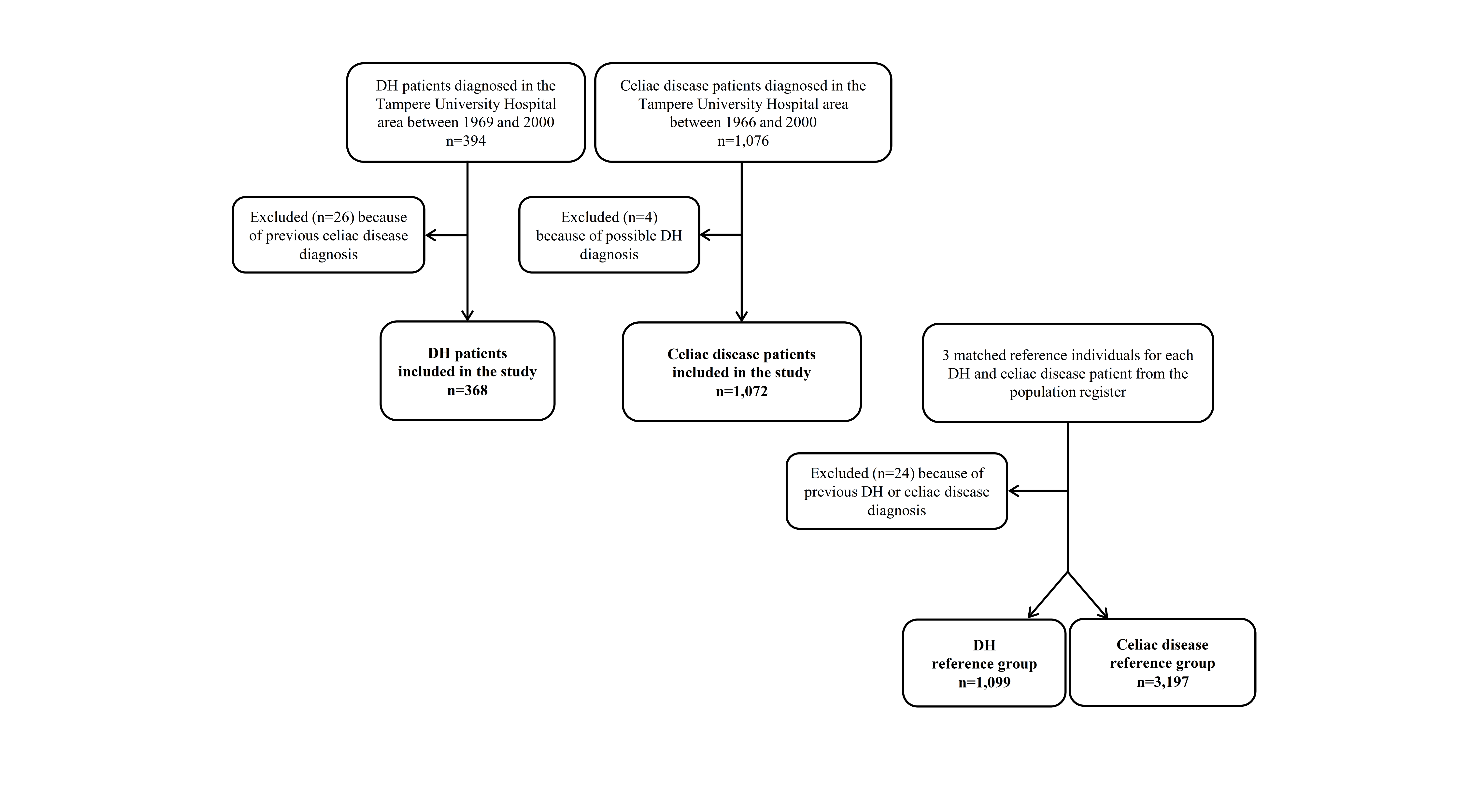

Supplement: Supplemental Material [file IANN_A_2227423_SM7135.png]
